# Sleep Disturbances and Sexual Dysfunction in Parkinson’s Disease: Sex Differences and Impact on Quality of Life in a Turkish Cohort

**DOI:** 10.3390/jcm15052065

**Published:** 2026-03-09

**Authors:** Burcu Gökçe Çokal, Bünyamin Tosunoğlu, Hatice Mediha Kına, Kübra Mehel Metin, Hafize Nalan Güneş

**Affiliations:** Department of Neurology, Ankara Training and Research Hospital, University of Health Sciences, Ankara 06230, Turkey

**Keywords:** Parkinson’s disease, sexual dysfunction, sleep quality, excessive daytime sleepiness, gender differences, quality of life

## Abstract

**Background**: Sexual dysfunction (SD) and sleep disturbances are frequent but underrecognized non-motor symptoms in Parkinson’s disease (PD) and significantly affect quality of life. However, the relationships among sexual dysfunction, sleep quality, and excessive daytime sleepiness (EDS) and the possible sex-related differences remain insufficiently investigated. **Methods**: In this cross-sectional case–control study, we evaluated these non-motor symptoms in 147 Turkish patients with PD and 160 age- and sex-matched healthy controls, and we assessed their associations and impact on quality of life, with particular attention to sex-specific patterns. Sexual function was assessed using the Arizona Sexual Experiences Scale (ASEX), sleep quality using the Pittsburgh Sleep Quality Index (PSQI), daytime sleepiness using the Epworth Sleepiness Scale (ESS), quality of life using the 39-item Parkinson’s Disease Questionnaire (PDQ-39), and disease severity using the Unified Parkinson’s Disease Rating Scale (UPDRS) and Hoehn and Yahr (H&Y) staging scale. Group comparisons, correlation analyses, and sex-stratified subgroup analyses were performed. **Results**: Patients with PD had significantly higher ASEX, PSQI, and ESS scores compared with controls (*p* < 0.01), and women with PD had significantly higher total ASEX scores than men, indicating greater sexual dysfunction. Sexual dysfunction was significantly associated with poor sleep quality and excessive daytime sleepiness but showed no significant association with the motor severity measures (UPDRS, H&Y stage). Sleep quality, as measured via PSQI scores, was worse in patients with PD, and poor sleep quality and excessive daytime sleepiness were both associated with significantly worse quality of life. **Conclusions**: According to our findings, sexual dysfunction and sleep disturbances are interrelated non-motor symptoms that significantly impair quality of life, largely independently of motor severity, and these associations were particularly pronounced among women. A combined evaluation of sleep and sexual function may therefore improve the recognition and management of the non-motor burden in PD.

## 1. Introduction

Parkinson’s disease (PD) is a progressive neurodegenerative disorder characterized by both motor and non-motor symptoms (NMSs) [1].

In addition to an α-synuclein pathology and dopaminergic neurodegeneration, neuroinflammatory, metabolic/mitochondrial, and environmental factors are increasingly being recognized as contributors to PD pathogenesis [2,3,4].

According to the Global Burden of Disease (GBD) 2021 estimates, 11.77 million people were living with PD worldwide, and this number is expected to more than double by 2050, primarily due to population aging [5]. NMSs such as sexual dysfunction, sleep disturbances, neuropsychiatric features, and autonomic impairment are highly prevalent in PD and contribute to quality of life as much as motor features [6]. Among autonomic non-motor symptoms, sexual dysfunction is highly prevalent in PD but remains underrecognized in routine clinical practice, partly due to misconceptions and cultural biases [7]. In studies investigating the prevalence of sexual dysfunction in PD, the reported rates range from 42% to 79% in men and from 36% to 87.5% in women. This variability is thought to result from heterogeneity in definitions, patient cohorts, and assessment tools, as well as from the multifactorial etiology of sexual dysfunction involving neurodegenerative, autonomic, psychological, and iatrogenic factors [8,9]. Sleep disturbances are a common feature of PD, occurring in up to 90% of patients throughout the disease course, and conditions including insomnia, REM sleep behavior disorder, restless legs syndrome (RLS), and EDS are frequently observed and typically worsen as the disease progresses [10].

Sleep disturbances are increasingly being recognized as a prominent non-motor feature across the parkinsonian spectrum, extending beyond Parkinson’s disease to atypical parkinsonian disorders such as progressive supranuclear palsy syndrome (PSPS) and corticobasal syndrome (CBS), where a high prevalence of sleep–wake abnormalities has been reported, including excessive daytime sleepiness and nocturnal sleep fragmentation [11].

Although clinically important, sexual dysfunction, sleep quality, and excessive daytime sleepiness (EDS) have mostly been examined individually rather than in combination, and no study to date has evaluated these three non-motor symptoms together in a Turkish PD cohort with a specific focus on sex-related differences.

Previous case–control studies from non-Western populations, including Middle Eastern and Turkish cohorts, have reported a high prevalence of sexual dysfunction and its detrimental impact on quality of life in patients with PD [12,13,14]. Nevertheless, these studies largely focused on sexual dysfunction in isolation and did not comprehensively explore its relationships with sleep quality and excessive daytime sleepiness. Similarly, the international literature has highlighted substantial burdens of both sexual dysfunction and sleep disturbances in PD and their negative effects on quality of life; however, these symptom domains have generally been investigated separately rather than within an integrated framework [15,16,17].

Therefore, the present study aimed to evaluate sexual dysfunction together with sleep quality and excessive daytime sleepiness and examine their combined associations with quality of life in a Turkish cohort of patients with PD. We hypothesized that these non-motor symptoms would be independently associated with reduced quality of life regardless of motor severity, and that the magnitude of these associations might differ by sex.

## 2. Materials and Methods

A total of 147 patients with idiopathic PD, diagnosed according to the UK Parkinson’s Disease Society Brain Bank criteria, who presented to the Movement Disorders Outpatient Clinic, Department of Neurology, University of Health Sciences Ankara Training and Research Hospital, between May 2025 and August 2025 were enrolled in this study, as well as 160 age- and sex-matched healthy controls. Ethical approval was obtained from the institutional Ethics Committee (Approval No.: E–25–442), and all participants provided written informed consent. Patients were excluded if they had dementia (Mini-Mental State Examination [MMSE] < 24), secondary parkinsonism or parkinsonism-plus syndromes, Hoehn and Yahr stage 5 disease, severe sensory or neurological impairments interfering with assessment, chronic obstructive pulmonary disease, urological disorders, alcohol or substance abuse, or a history of psychiatric disorders. Psychiatric conditions, including depression and anxiety, were screened through medical record review and direct patient interviews, including history of psychiatric diagnoses and psychotropic medication use. Patients who had used sedatives, hypnotics, antidepressants, or opioids within the previous 6 months were also excluded, as well as those with medication adjustments within the four weeks preceding enrollment.

All patients with Parkinson’s disease were receiving stable antiparkinsonian pharmacotherapy at the time of assessment and were categorized according to their treatment regimens (dopamine agonist therapy, levodopa therapy, rasagiline monotherapy, or levodopa-plus-dopamine agonist therapy).

The motor severity and disease stage were evaluated with the Movement Disorder Society-sponsored revision of the Unified Parkinson’s Disease Rating Scale (UPDRS) [18] and the modified H&Y staging scale [19]. Sleep quality was assessed using the Pittsburgh Sleep Quality Index (PSQI), a 19-item self-administered questionnaire yielding a global score from 0 to 21, with higher scores indicating poorer sleep quality; patients were classified as good (PSQI ≤ 5) or poor (PSQI > 5) sleepers [20,21]. Daytime sleepiness was measured with the Epworth Sleepiness Scale (ESS), an 8-item self-report questionnaire with scores ranging from 0 to 24, where higher scores reflect greater sleep propensity [22,23].

Excessive daytime sleepiness (EDS) was defined as an ESS score > 10. Sexual function was assessed using the Arizona Sexual Experiences Scale (ASEX), a five-item questionnaire evaluating sexual desire, arousal, erection/lubrication, ability to achieve orgasm, and orgasmic satisfaction. Each item is scored from 1 to 6, yielding a total score ranging from 5 to 30, with higher scores indicating greater severity of sexual dysfunction [24,25].

Quality of life was assessed with the 39-item Parkinson’s Disease Questionnaire (PDQ-39), which yields a summary index and eight domain scores (mobility, activities of daily living, emotional well-being, stigma, social support, cognition, communication, and bodily discomfort), with higher scores reflecting poorer quality of life [26,27]. Sociodemographic data were collected for all participants, including age, sex, education level, employment status, and income level, and for patients with PD, the disease duration, levodopa equivalent daily dose (LEDD), Hoehn and Yahr stage, and Unified Parkinson’s Disease Rating Scale (UPDRS) scores were also recorded. The total UPDRS score was calculated as the sum of Parts I–III, while Part IV was recorded separately. Health-related quality of life was assessed using the PDQ-39 summary index. In addition, subgroup analyses within the PD group were performed to compare sexual function, sleep quality, and excessive daytime sleepiness according to the dopaminergic treatment type (dopamine agonists or levodopa, with or without rasagiline).

### Statistical Analysis

All statistical analyses were performed using SPSS for Windows, version 22.0 (SPSS Inc., Chicago, IL, USA). As most variables did not meet normality assumptions, nonparametric tests were applied. Categorical variables are presented as n (%), and continuous variables as median (interquartile range (IQR): 25th–75th percentile). Between-group comparisons were conducted using the Mann–Whitney U test for continuous variables involving two groups and the Kruskal–Wallis test for comparisons across more than two groups. Categorical variables were analyzed using the chi-square test (or Fisher’s exact test where appropriate). When the Kruskal–Wallis test indicated a significant difference, post hoc pairwise comparisons were performed using Bonferroni-corrected Mann–Whitney U tests. Associations between quantitative variables were examined using Spearman’s rank correlation coefficient (Spearman’s r), with a two-tailed *p*-value < 0.05 considered statistically significant. The correlation matrix was visualized using a heatmap to provide a clearer overview of the associations among the clinical and demographic variables. The correlation strength was interpreted according to conventional criteria, with correlation coefficients of 0.10–0.29 considered weak, those of 0.30–0.49 considered moderate, and those ≥0.50 considered strong.

To evaluate independent predictors of quality of life (total PDQ-39 score), multivariable linear regression models were constructed including age, sex, income level, total UPDRS score, and disease duration as covariates. Robust (HC3) standard errors were applied to account for potential heteroskedasticity. Multicollinearity was assessed using variance inflation factors (VIFs), and model assumptions were evaluated through residual diagnostics. Parallel mediation analyses were conducted to examine whether sexual dysfunction (total ASEX score) and sleep quality (total PSQI score) mediated the association between sex and quality of life. The mediation models were adjusted for the same covariates (age, income level, total UPDRS score, and disease duration), and the significance of indirect effects was tested using the Sobel test. Detailed model diagnostics and additional statistical outputs are provided in the Supplementary Materials (Tables S1–S3).

## 3. Results

No significant differences were observed between the groups in terms of age (*p* = 0.122) and sex distribution (*p* = 0.070; Table 1). Patients with PD showed significantly worse sexual function (total ASEX and all subdomains, all *p* ≤ 0.001) and higher ESS (median 7 vs. 2, *p* < 0.001) and PSQI (*p* = 0.002) scores, and excessive daytime sleepiness was more common among them (29.3% vs. 6.3%, *p* < 0.001) (Table 2).

Although the total PSQI scores were significantly higher in patients with PD, the proportions of individuals exceeding the conventional cut-off (>5) did not differ significantly between groups.

The proportion of poor sleepers (PSQI > 5) was higher in patients with PD (57.1% vs. 48.1%); however, this difference was not statistically significant (*p* = 0.114) (Table 2). The median disease duration was 4 years (IQR: 3–6), the LEDD was 400 mg/day (IQR: 100–400), and the median Hoehn and Yahr stage was 2 (IQR: 1.5–2.5). Moreover, the median PDQ-39 summary index was 35.26 (IQR: 24–49), the median total UPDRS score (Parts I–III) was 15 (IQR: 11–24), with median subscores of 1 (IQR: 1–3), 7 (IQR: 7–9), and 7 (IQR: 7–12) for Parts I, II, and III, respectively, and motor complications were minimal, with a median UPDRS Part IV score of 0 (IQR: 0–0) (Table 3).

The total ASEX scores correlated with the ESS (r = 0.385, *p* < 0.001), PSQI (r = 0.440, *p* < 0.001), and PDQ-39 (r = 0.538, *p* < 0.001), and a significant correlation with age was also observed (r = 0.219, *p* = 0.008). The ASEX scores were not associated with the motor severity measures (UPDRS and H&Y stage), nor with the LEDD.

**Figure 1:**
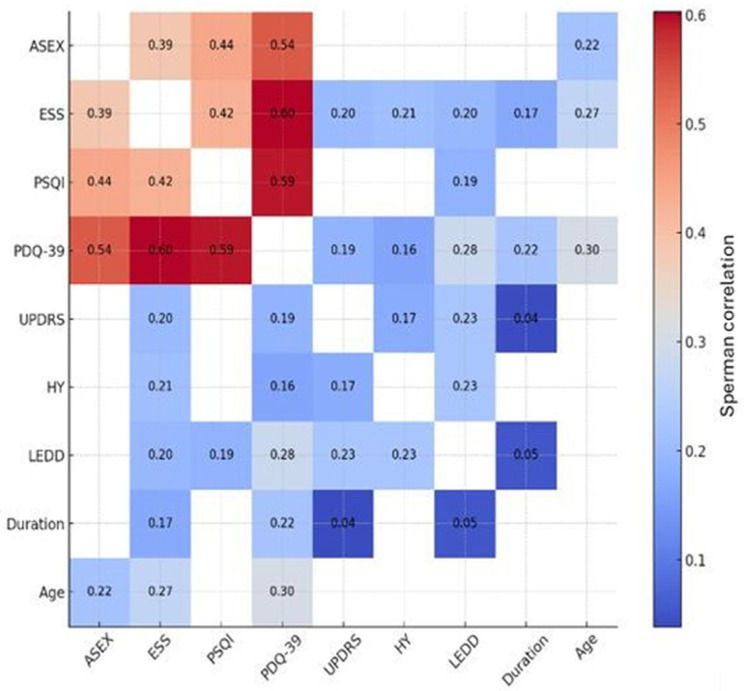
Spearman correlation heatmap of clinical and demographic variables in Parkinson’s disease patients.

The ESS scores correlated with the PSQI (r = 0.425, *p* < 0.001), PDQ-39 (r = 0.603, *p* < 0.001), and clinical severity measures, including age and the UPDRS, Hoehn and Yahr stage, LEDD, and disease duration; the PSQI scores correlated with the PDQ-39 (r = 0.593, *p* < 0.001) and LEDD (r = 0.186, *p* = 0.024); and the PDQ-39 scores were also associated with age and the UPDRS, H&Y stage, LEDD, and disease duration (Figure 1).

Multivariable analyses:

In the multivariable linear regression analyses, longer disease duration, higher sexual dysfunction (ASEX), greater excessive daytime sleepiness (ESS), and poorer sleep quality (PSQI) were independently associated with worse quality of life (total PDQ-39 score). In contrast, age, sex, income level, and motor severity (total UPDRS score) were not independently associated with quality of life in the adjusted model (Table 4).

Exploratory analyses of socioeconomic factors:

Kruskal–Wallis tests revealed significant differences in sexual dysfunction (ASEX) (χ^2^ = 10.16, *p* = 0.006) and daytime sleepiness (ESS) (χ^2^ = 6.88, *p* = 0.032) across income groups. Post hoc Bonferroni-corrected Mann–Whitney U tests indicated that the ASEX difference was primarily driven by significantly higher scores in the low-income group compared to the high-income group (*p* = 0.007), whereas differences between the low- and middle-income groups did not remain statistically significant after correction. Pairwise differences for ESS were less consistent and did not remain statistically significant after Bonferroni correction.

Although the income level differed significantly between groups and was associated with sexual dysfunction and daytime sleepiness in the exploratory analyses, it was not independently associated with quality of life in the adjusted models. Importantly, the mediation pathway linking sex to quality of life via sexual dysfunction and sleep quality remained statistically significant after controlling for income (Supplementary Tables S2 and S3).

β coefficients are presented with robust (HC3) standard errors. Model R^2^ = 0.662 (adjusted R^2^ = 0.640). Higher PDQ-39 scores indicate poorer quality of life. UPDRS: Unified Parkinson’s Disease Rating Scale; ASEX: Arizona Sexual Experiences Scale; ESS: Epworth Sleepiness Scale; PSQI: Pittsburgh Sleep Quality Index.

Patients with poor sleep quality exhibited significantly worse quality of life and higher total ASEX, subdomain, and ESS scores (all *p* ≤ 0.001). Age, LEDDs, UPDRS scores, and H&Y staging did not differ between good and poor sleepers; however, women were more common in the group with poor sleep quality (Table 5). Excessive daytime sleepiness was present in 29.3% of patients with PD, and compared with patients without excessive daytime sleepiness (ESS ≤ 10), these patients were older and showed worse quality of life, greater motor severity, higher ASEX scores across all domains, and poorer sleep quality (all *p* < 0.05) (Table 6).

In both control participants and patients with PD, women had significantly higher total ASEX and subdomain scores than men (all *p* < 0.001), while no sex differences were observed in the excessive daytime sleepiness prevalence or ESS scores. Moreover, women with PD had poorer sleep quality, with higher PSQI scores, and were more frequently classified as poor sleepers (PSQI > 5) (Table 7). The distribution of antiparkinsonian medications in the PD cohort was as follows: dopamine agonist therapy in 50 patients (34.0%), levodopa therapy in 70 patients (47.6%), rasagiline monotherapy in 12 patients (8.2%), and levodopa-plus-dopamine agonist therapy in 15 patients (10.2%). A comparison according to dopaminergic treatment (dopamine agonists or levodopa, with or without rasagiline) showed no significant differences in sexual function, sleep quality, or excessive daytime sleepiness. Patients receiving levodopa had higher motor severity and more advanced disease; however, no significant differences were observed between treatment groups in quality of life, sleep quality, or sexual function. These analyses are presented in the Supplementary Materials (Supplementary Table S4).

## 4. Discussion

Sexual dysfunction was markedly more severe in patients with PD than in healthy controls, with significant impairment across all ASEX subdomains, indicating a multidimensional disturbance involving sexual desire, arousal, and orgasmic function. This finding is consistent with previous ASEX-based studies demonstrating increased sexual dysfunction in patients with PD across different populations [9]. The ASEX scores were not related to the motor severity, disease stage, or levodopa dose, suggesting that sexual dysfunction in PD occurs largely independently of motor impairment. This observation aligns with prior research showing stronger associations between sexual dysfunction and quality of life than with motor burden alone [28], as well as with age-related influences [13]. We did not observe the associations with motor severity reported by Jitkritsadakul et al. [9] in our cohort, which may reflect differences in the disease stage distributions, assessment methodologies, and sample characteristics.

From a pathophysiological perspective, sexual dysfunction in PD appears to be primarily related to non-motor mechanisms, including autonomic and limbic system dysfunction and the altered dopaminergic regulation of reward processing. A shared α-synuclein pathology within the brainstem and limbic circuits has been proposed as a potential explanation for the frequent coexistence of sexual and sleep disturbances, and increased α-synuclein deposition in regions regulating the sleep–wake cycle, such as the locus coeruleus, raphe nuclei, posterior hypothalamus, and thalamus, has been described in PD patients with sleep disorders [29,30]. Because these structures are also involved in emotional and sexual regulation, overlapping neurodegenerative processes may underlie the co-occurrence of sexual dysfunction, poor sleep quality, and excessive daytime sleepiness.

Excessive daytime sleepiness was present in 29.3% of patients with PD, a prevalence consistent with systematic reviews and meta-analyses reporting rates affecting approximately one-quarter to one-third of patients [31,32]. Given that the median Hoehn and Yahr stage in our cohort was 2, this prevalence may reflect an early-to-moderate disease population, as previous studies have shown that excessive daytime sleepiness tends to increase with advancing disease severity. Although the ESS scores were numerically higher in men, this difference was not statistically significant. Previous studies have yielded heterogeneous findings regarding sex differences in excessive daytime sleepiness, with some reporting no difference [32] and others suggesting greater vulnerability in men [33]. These discrepancies indicate that excessive daytime sleepiness likely reflects a multifactorial process influenced by age, disease duration, neurodegenerative burden, comorbidities, and treatment factors rather than sex alone [34].

An additional finding warranting consideration is the discrepancy between the continuous total PSQI scores and the categorical classification of poor sleepers. Although patients with PD exhibited significantly higher total PSQI scores, the proportions exceeding the conventional cut-off (>5) did not differ significantly between groups, a pattern also reported by Pezzini et al. [31], which suggests that the sole reliance on threshold-based categorization may underestimate the clinically meaningful sleep-related symptom burden. Sleep disturbances in PD are heterogeneous and encompass insomnia, REM sleep behavior disorder, nocturnal motor symptoms, and medication-related effects, which may not be adequately captured by a single global PSQI cut-off. Consistent with this interpretation, systematic reviews and meta-analyses have demonstrated substantial variability in their reported prevalence rates across PD cohorts [35,36]. In both research and clinical practice, a nuanced interpretation of continuous-scale scores may therefore provide a more accurate reflection of sleep disturbance severity than rigid dichotomous thresholds.

When considered within the broader international literature, our findings align with observations from larger European and multicenter cohorts demonstrating a substantial burden and clustering of non-motor symptoms in PD. Previous studies have shown that both sleep disturbances and sexual dysfunction independently contribute to reduced quality of life in PD populations [15,16,17], and comprehensive analyses further emphasize the cumulative and interactive impact of coexisting non-motor symptoms on health-related quality of life. By jointly evaluating sexual dysfunction, sleep quality, and excessive daytime sleepiness within a single cohort, our study provides an integrated perspective on the non-motor symptom interplay and extends the existing literature, in which these domains have largely been examined separately.

However, differences in prevalence estimates and effect sizes across studies likely reflect heterogeneity in the cohort compositions, disease stage distributions, medication profiles, and assessment instruments, as well as cultural and healthcare system-related factors that may influence symptom reporting, particularly for sensitive domains such as sexual health.

The total ASEX scores were associated with sleep quality, daytime sleepiness, and quality of life but not with motor severity. This suggests that sexual dysfunction and sleep problems tend to co-occur as non-motor symptoms and are jointly associated with poorer quality of life in PD. Importantly, multivariable analyses confirmed that sexual dysfunction, poor sleep quality, and excessive daytime sleepiness were independently associated with worse quality of life after adjustment for motor severity and demographic factors, underscoring that the non-motor symptom burden contributes to quality-of-life impairment beyond the effects of motor involvement alone.

With respect to sex-related differences, sleep quality did not differ between men and women in the control group, whereas women with PD reported poorer sleep quality and were more frequently classified as poor sleepers. This pattern is consistent with findings from large cohorts, including the French CoPark study, which demonstrated a higher burden of sleep disturbances among women with PD [37].

The magnitude of the sex-related differences observed in our single-center Turkish sample may differ from that observed in larger multicenter, international cohorts due to differences in the sociodemographic compositions, cultural contexts, and health-seeking behaviors, as well as methodological differences across studies, which limits direct cross-study comparability. However, other studies have reported poorer sleep quality in men or no significant sex differences, highlighting the heterogeneity of the findings and the potential influence of the sample characteristics and sociocultural context [15,38]. Although excessive daytime sleepiness did not differ significantly between the sexes in our cohort, conflicting evidence persists regarding sex-related effects on sleep–wake disturbances [39]. Sexual dysfunction was more pronounced in women in both the PD and control groups. While we did not assess hormonal or neurobiological parameters, the previous literature has suggested that such sex-related differences may reflect a combination of biological, neuropathological, and sociocultural influences. For instance, cultural norms and taboos surrounding sexuality may limit symptom disclosure and help-seeking behavior among women [40]. In addition, it has been proposed that mechanisms such as estrogen-mediated neuroprotection, differential dopaminergic vulnerability, and sex-specific patterns of limbic system involvement contribute to the expression of non-motor symptoms in PD [1,41], and neurodegenerative changes in limbic and hypothalamic nuclei, as well as in peripheral autonomic structures, have also been implicated in the pathophysiology of sexual dysfunction [8]. These hypotheses, while not directly addressed in our study, may help contextualize our sex-related findings.

Moreover, we observed no significant differences in sexual function, sleep quality, excessive daytime sleepiness, or quality of life across the different dopaminergic treatment regimens. Although dopamine agonists have been linked to impulse control disorders and excessive daytime sleepiness [42,43], the current evidence regarding their independent impact on sleep and sexual function remains inconclusive.

Several studies have reported no significant association between different dopaminergic treatments and excessive daytime sleepiness after adjustment for disease severity and other clinical factors [43]. Regarding sexual function, while the association of dopamine agonists with hypersexuality and other impulse control disorders is well known, a direct relationship between dopaminergic treatment and sexual dysfunction as assessed via validated instruments such as the ASEX has not been consistently demonstrated [8,43].

Overall, these findings suggest that sleep disturbances and sexual dysfunction in PD are more closely related to disease-specific non-motor mechanisms than to the type of dopaminergic therapy.

Although income level was not independently associated with overall quality of life after adjustment for clinical and demographic variables, exploratory analyses suggested greater sexual dysfunction and excessive daytime sleepiness in the lowest-income group. However, these findings should be interpreted cautiously given the markedly unequal distribution of income strata. Socioeconomic disadvantage may plausibly influence the non-motor symptom burden through disparities in healthcare access and health literacy and barriers to reporting sensitive symptoms [44,45].

Importantly, the indirect effect of sex on quality of life via sexual dysfunction and sleep quality remained significant after controlling for income, indicating that these non-motor symptoms represent core contributors to quality-of-life impairment independent of socioeconomic status.

Future studies with more socioeconomically balanced samples are warranted to further clarify the role of social determinants in the non-motor symptom burden in PD.

Our findings indicate that longer disease duration is independently associated with poorer quality of life in patients with Parkinson’s disease. This observation is consistent with previous studies demonstrating that advancing disease is accompanied by increasing disability and emotional distress and reduced social participation [46,47,48].

Notably, prior research has shown that disease duration remains significantly associated with lower health-related quality of life even after adjustment for age and motor severity, highlighting its contribution beyond purely motor progression [46,48]. Collectively, these findings underscore that disease duration represents a clinically meaningful determinant of perceived well-being in PD populations.

This study has several limitations that should be acknowledged. First, its cross-sectional design precludes causal or directional inferences regarding the relationships between sexual dysfunction, sleep disturbances, and quality of life. Second, the relatively small sample size may limit the generalizability of the findings, particularly for subgroup analyses. Third, although income level was included as a covariate in the multivariable and mediation models, the markedly unequal distribution of income groups may limit the robustness of these estimates. Therefore, the socioeconomic findings should be interpreted cautiously. Fourth, medical comorbidities were not systematically assessed; therefore, their potential impact on sleep-related outcomes cannot be fully excluded. Fifth, depressive and anxiety symptoms were not evaluated using standardized rating scales; therefore, subclinical mood symptoms and their potential confounding effects cannot be fully excluded, representing an important limitation of the present study given the documented associations between sexual dysfunction, depression, and anxiety in Parkinson’s disease [49]. Finally, the reliance on self-reported questionnaires may have introduced recall and reporting bias, and objective sleep assessments, such as polysomnography, were not performed.

Despite these limitations, our study has notable strengths, including a multidimensional assessment of sexual dysfunction, sleep quality, and daytime sleepiness in patients with PD, with additional subgroup and sex-based analyses, which have rarely been addressed in the literature.

To the best of our knowledge, no previous study on a Turkish cohort has examined sexual dysfunction together with sleep quality and excessive daytime sleepiness in PD. Our study demonstrated that sexual dysfunction was more severe in women, as reflected by the higher ASEX scores in both the PD and control groups, and that female patients with PD experienced poorer sleep quality compared with men. Although excessive daytime sleepiness did not differ by sex, sexual dysfunction was strongly associated with poor sleep quality and daytime sleepiness, particularly in women, and these non-motor symptoms were strongly associated with poorer quality of life, largely independent of motor severity. However, given the cross-sectional nature of this study, the observed relationships should be interpreted as associative rather than as indicative of causal direction.

## 5. Conclusions

Our findings highlight the importance of incorporating sex-related differences into clinical evaluations and support a more comprehensive, gender-sensitive approach to the assessment of non-motor symptoms in Parkinson’s disease. Sexual dysfunction, poor sleep quality, and excessive daytime sleepiness were found to be strongly associated with impaired quality of life, largely independent of motor severity.

These results underscore that the non-motor burden cannot be inferred from motor scales alone and support the need for routine, structured screening in everyday neurological practice. From a practical perspective, reports of poor sleep quality or excessive daytime sleepiness should prompt a broader evaluation of non-motor symptoms, including sexual health.

Further large-scale prospective studies are warranted to clarify the underlying mechanisms and temporal relationships.

## Figures and Tables

**Table 1:** Demographic characteristics of control and Parkinson’s disease groups.

Variable	Control (n = 160)	Parkinson’s Disease (n = 147)	*p*
Age, years	69 (64–72)	70 (66–74)	0.122 *
Sex, n (%)			0.070 **
Female	84 (52.5)	61 (41.5)	
Male	76 (47.5)	86 (58.5)	
Education Level, n (%)			0.002 **
University	16 (10.0)	8 (5.4)	
High school	31 (19.4)	22 (15.0)	
Secondary school	33 (20.6)	62 (42.2)	
Primary school	60 (37.5)	38 (25.9)	
Literate (without formal education)	10 (6.3)	11 (7.5)	
Illiterate (without formal education)	10 (6.3)	6 (4.1)	
Marital Status, n (%)			0.305 **
Married	123 (76.9)	120 (81.6)	
Single/divorced	37 (23.1)	27 (18.4)	
Employment Status, n (%)			0.415 **
Employed	27 (16.9)	17 (11.6)	
Retired	76 (47.5)	74 (50.3)	
Unemployed	57 (35.6)	56 (38.1)	
Income Status, n (%)			<0.001 **
Low	90 (56.3)	8 (5.4)	
Medium	65 (40.6)	126 (85.7)	
High	5 (3.1)	13 (8.8)	

* Mann–Whitney U test; ** chi-square test. Categorical variables are presented as n (%).

**Table 2:** Comparison of sexual function, sleep quality, and daytime sleepiness between PD patients and controls.

Variable	Parkinson’s Disease (n = 147)	Control (n = 160)	*p*
Total ASEX Score	22 (16–26)	17 (14–20)	<0.001 *
ASEX-1 (sexual drive)	4 (3–5)	3 (3–4)	0.001 *
ASEX-2 (arousal)	5 (3–5)	4 (3–5)	0.001 *
ASEX-3 (penile erection/vaginal lubrication)	5 (4–6)	4 (3–5)	<0.001 *
ASEX-4 (ability to orgasm)	5 (3–5)	4 (2–5)	<0.001 *
ASEX-5 (satisfaction)	4 (3–5)	3 (2–4)	<0.001 *
ESS, n (%)			<0.001 **
0–10	104 (70.7)	150 (93.8)	
>10	43 (29.3)	10 (6.3)	
Total ESS Score	7 (3–11)	2 (1–5)	<0.001 *
Total PSQI Score	6 (4–10)	5 (3–7)	0.002 *
PSQI, n (%)			0.114 **
Good (≤5)	63 (42.9)	83 (51.9)	
Poor (>5)	84 (57.1)	77 (48.1)	

* Mann–Whitney U test; ** chi-square test. Categorical variables are presented as n (%), and continuous variables are presented as median (interquartile range (IQR)). ASEX: Arizona Sexual Experiences Scale; ESS: Epworth Sleepiness Scale; PSQI: Pittsburgh Sleep Quality Index.

**Table 3:** Clinical characteristics of patients with Parkinson’s disease.

Variable	
Disease Duration (years)	4 (3–6)
LEDD (mg/day)	400 (100–400)
H&Y	2.0 (1.5–2.5)
PDQ-39 Summary Index	35.26 (24–49)
Mobility	37.5 (20–60)
Activities of Daily Living	45.8 (29.2–62.5)
Emotional Well-Being	41.7 (25–62.5)
Stigma	0 (0–25)
Social Support	0 (0–16.7)
Cognition	50 (31.25–68.75)
Communication	16.7 (8.33–41.7)
Bodily Discomfort	25 (8.3–58.3)
Total UPDRS Score (I–IV)	15 (11–24)
UPDRS I	1 (1–3)
UPDRS II	7 (7–9)
UPDRS III	7 (7–12)
UPDRS IV	0 (0–0)

Values are presented as median (interquartile range (IQR)). LEDD: levodopa equivalent daily dose; H&Y: Hoehn and Yahr stage; PDQ-39: Parkinson’s Disease Questionnaire; UPDRS: Unified Parkinson’s Disease Rating Scale.

**Table 4:** Multivariable linear regression analysis of factors associated with quality of life (total PDQ-39 score).

Predictor	β (SE)	*p*-Value
Age (years)	0.13 (0.27)	0.616
Sex (female vs. male)	2.12 (4.61)	0.646
Income (medium vs. low)	2.33 (8.11)	0.774
Income (high vs. low)	−0.89 (6.47)	0.891
Total UPDRS Score	−0.04 (0.10)	0.688
Disease Duration (years)	2.48 (0.84)	0.003
Total ASEX Score	1.01 (0.40)	0.012
Total ESS Score	1.93 (0.44)	<0.001
Total PSQI Score	2.64 (0.51)	<0.001

**Table 5:** Clinical characteristics of Parkinson’s disease patients by sleep quality.

Variable	Good Sleep (PSQI ≤ 5) n = 63	Poor Sleep (PSQI > 5) n = 84	*p*
Age (years)	68 (64–72)	68 (65–73)	0.691 *
Sex, n (%)			
Female	19 (30.2)	42 (50.0)	0.025 **
Male	44 (69.8)	42 (50.0)	
LEDD (mg/day)	300 (100–400)	400 (141–425)	0.173 *
PDQ-39 summary index	29.5 (18.36–38.5)	42.3 (30.3–58.0)	<0.001 *
Total UPDRS score (I–IV)	15 (15–25)	15 (15–25)	0.421 *
UPDRS I	1 (1–3)	1 (1–3)	0.988 *
UPDRS II	7 (7–9)	7 (7–10.75)	0.515 *
UPDRS III	7 (7–12)	7 (7–12)	0.338 *
UPDRS IV	0 (0–0)	0 (0–0)	0.198 *
H&Y	2 (1.5–2.5)	2 (1.5–2.5)	0.721 *
Total ASEX score	17 (14–21)	20 (16–26)	0.001 *
ASEX-1 (sexual drive)	3 (3–4)	4 (3–5)	0.001 *
ASEX-2 (arousal)	4 (3–5)	5 (3–5)	0.001 *
ASEX-3 (penile erection/vaginal lubrication)	4 (3–5)	5 (3–6)	0.001 *
ASEX-4 (ability to orgasm)	4 (2–5)	5 (2–6)	0.001 *
ASEX-5 (satisfaction)	3 (2–5)	4 (2–5)	0.004 *
Total ESS score	2 (1–5)	6 (3–11)	<0.001 *

* Mann–Whitney U test; ** chi–square test. Continuous variables are presented as median (interquartile range (IQR)). LEDD: levodopa equivalent daily dose; PDQ-39: Parkinson’s Disease Questionnaire; UPDRS: Unified Parkinson’s Disease Rating Scale; H&Y: Hoehn and Yahr stage; ASEX: Arizona Sexual Experiences Scale; ESS: Epworth Sleepiness Scale.

**Table 6:** Clinical characteristics of Parkinson’s disease patients by daytime sleepiness status.

Variables	Normal (ESS: 0–10) (n = 104)	EDS (ESS > 10) (n = 43)	*p*
Age	69 (65–73)	72 (69–75)	0.014 *
Sex, n (%)			
Female	41 (39.4)	20 (46.5)	0.542 **
Male	63 (60.6)	23 (53.5)	
LEDD (mg/day)	387 (100–400)	400 (138–525)	0.081 *
PDQ-39 summary index	30.8 (20.8–41.5)	57.05 (41.67–75)	<0.001 *
Total UPDRS score (I–IV)	15 (13.5–25)	15 (15–40)	0.012 *
UPDRS I	1 (0–2)	1 (1–4)	0.002 *
UPDRS II	7 (5.25–9)	7 (7–19)	0.010 *
UPDRS III	7 (7–12)	7 (7–12)	0.045 *
UPDRS IV	0 (0–0)	0 (0–0)	0.474
H&Y	2 (1–2)	2 (1.5–3)	0.008 *
Total ASEX score	18 (14–24)	24 (18–30)	<0.001 *
ASEX-1 (sexual drive)	4 (3–5)	5 (3–6)	<0.001 *
ASEX-2 (arousal)	4 (3–5)	5 (5–6)	<0.001 *
ASEX-3 (penile erection/vaginal lubrication)	5 (3–5)	6 (5–6)	<0.001 *
ASEX-4 (ability to orgasm)	5 (2.25–5)	6 (4–6)	<0.001 *
ASEX-5 (satisfaction)	4 (3–5)	5 (4–6)	<0.001 *
Total PSQI score	5 (4–8)	11 (6–15)	<0.001 *

* Mann–Whitney U test; ** chi-square test. Categorical variables are presented as n (%), and continuous variables as median (interquartile range (IQR)). EDS: excessive daytime sleepiness (EDS: ESS score > 10; normal: ESS score: 0–10); LEDD: levodopa equivalent daily dose; PDQ-39: Parkinson’s Disease Questionnaire; UPDRS: Unified Parkinson’s Disease Rating Scale; H&Y: Hoehn and Yahr stage; ASEX: Arizona Sexual Experiences Scale; PSQI: Pittsburgh Sleep Quality Index.

**Table 7:** Subgroup analysis by sex in control and Parkinson’s disease groups.

Variable	Control			PD		
	Female (n = 84)	Male (n = 76)	*p*	Female (n = 61)	Male (n = 86)	*p*
Total ASEX score	20 (17–25.75)	14 (11–16)	<0.001 *	26 (24–29)	17 (13.75–22.25)	<0.001 *
ASEX-1 (sexual drive)	4 (3–5)	3 (2–3)	<0.001 *	5 (4–6)	3 (2–4)	<0.001 *
ASEX-2 (arousal)	4 (4–5)	3 (2–4)	<0.001 *	5 (5–6)	3.5 (2–5)	<0.001 *
ASEX-3 (penile erection/vaginal lubrication)	4 (3–5)	4 (2–4)	<0.001 *	5 (5–6)	5 (3–5)	<0.001 *
ASEX-4 (ability to orgasm)	5 (4–5.75)	2 (2–3)	<0.001 *	5 (5–6)	3.5 (2–5)	<0.001 *
ASEX-5 (satisfaction)	4 (3–5.75)	2 (2–3)	<0.001 *	5 (5–6)	3 (2–5)	<0.001 *
ESS, n (%)			0.624 **			0.542 **
0–10	80 (95.2)	70 (92.1)		41 (67.2)	63 (73.3)	
>10	4 (4.8)	6 (7.9)		20 (32.8)	23 (26.7)	
Total ESS score	2 (1–5)	2 (1–5.75)	0.798 *	7 (2.5–11.5)	8 (5–11)	0.948 *
Total PSQI score	5 (3–7)	5.5 (3–7)	0.646 *	6 (3.75–11.25)	5 (3.75–8.0)	0.002 *
PSQI, n (%)			0.652 **			0.025 **
Good (≤5)	45 (53.6)	38 (50.0)		19 (31.1)	44 (51.2)	
Poor (>5)	39 (46.4)	38 (50.0)		42 (68.9)	42 (48.8)	

* Mann–Whitney U test; ** chi–square test. Categorical variables are presented as n (%), and continuous variables are presented as median (interquartile range (IQR)). PD: Parkinson’s disease; ASEX: Arizona Sexual Experiences Scale; ESS: Epworth Sleepiness Scale; PSQI: Pittsburgh Sleep Quality Index.

## Data Availability

The datasets generated and/or analyzed during the current study are available from the corresponding author upon reasonable request.

## Supplementary Materials

The following supporting information can be downloaded at: https://www.mdpi.com/article/10.3390/jcm15052065/s1, Table S1: Multicollinearity diagnostics for the multivariable regression model; Table S2: Regression model diagnostic statistics; Table S3: Parallel mediation analysis examining the indirect effect of sex on quality of life (PDQ-39 total score); Table S4: Comparison of sleep and sexual function related variables according to dopaminergic treatment.
