# Supplementary material for: Sleep Disturbances and Sexual Dysfunction in Parkinson’s Disease: Sex Differences and Impact on Quality of Life in a Turkish Cohort
Source: J Clin Med. 2026 Mar 9;15(5):2065. doi: 10.3390/jcm15052065 (PMC12986440; doi:10.3390/jcm15052065)
Supplement: Supplementary file 1 [file jcm-15-02065-s001.zip › jcm-4161966-supplementary.pdf]

Journal of
Clinical Medicine

MDPI

Supplementary Materials

Supplementary Table S1. Multicollinearity Diagnostics for the Multivariable Regression Model.

Variable	VIF
Age	1.17
Sex	2.02
Income	1.09
UPDRS Total Score	1.15
Disease Duration	1.18
ASEX Total Score	2.37
ESS Total Score	1.79
PSQI Total Score	1.63

Residual diagnostics did not indicate violations of normality or independence assumptions. The condition number was 1.03×10^3. Although elevated, the low VIF values suggest that this likely reflects scaling differences among predictors rather than true multicollinearity. UPDRS: Unified Parkinson's Disease Rating Scale; ASEX: Arizona Sexual Experiences Scale; ESS: Epworth Sleepiness Scale; PSQI: Pittsburgh Sleep Quality Index.

All regression models were estimated using heteroskedasticity-robust (HC3) standard errors.

Supplementary Table S2. Regression Model Diagnostic Statistics.

Test	Statistic	*p*-Value
Omnibus	0.370	0.831
Jarque–Bera	0.486	0.784
Durbin–Watson	1.87	-

Residual diagnostics did not indicate violations of normality or independence assumptions. All regression models were estimated using robust (HC3) standard errors.

Supplementary Table S3. Parallel Mediation Analysis Examining the Indirect Effect of Sex on Quality of Life (PDQ-39 Total Score).

Path	Coefficient	*p*-Value
a_1: Sex → ASEX	8.28	<0.001
b_1: ASEX → PDQ-39	1.44	<0.001
Indirect effect (ASEX)	11.91	0.0008†
a_2: Sex → PSQI	2.48	<0.001
b_2: PSQI → PDQ-39	3.62	<0.001
Indirect effect (PSQI)	8.96	0.0012†
Direct effect (c′)	−2.19	0.646

Mediation analyses were performed using ordinary least squares regression with HC3 robust standard errors. All mediation models were adjusted for age, income level, UPDRS total score and disease duration.

 https://doi.org/10.3390/xxxxx

† p-values derived from Sobel test statistics (Z = 3.36 and Z = 3.24, respectively). Sobel tests confirmed the statistical significance of both indirect pathways ($p < 0.05$). PDQ-39: Parkinson's Disease Questionnaire; ASEX: Arizona Sexual Experiences Scale; PSQI: Pittsburgh Sleep Quality Index.

https://doi.org/10.3390/xxxxx

Supplementary Table S4. Comparison of sleep and sexual function related variables according to dopaminergic treatment.

Variable	Dopamine Agonist Group (n = 50)	Levodopa Group (n = 70)	*p*
Age, median (IQR 25–75)	69 (64–73)	70 (68–74)	0.232 *
Sex, n (%)			
Female	22 (44.0)	29 (41.4)	0.925 **
Male	28 (56.0)	41 (58.6)	
PDQ-39 Summary Index	30.77 (22.12–47.44)	36.86 (25.64–55.93)	0.296 *
UPDRS Total Score (I–IV)	15 (11–17)	15 (15–24)	0.009 *
H&Y	1.5 (1.0–2.0)	2 (1.5–2.5)	0.017 *
ASEX Total Score	20 (16–25)	22 (17–27)	0.486 *
ASEX-1 (Sexual drive)	4 (3–5)	4 (3–5)	0.791 *
ASEX-2 (Arousal)	4 (3–5)	5 (3.75–6)	0.100 *
ASEX-3 (Penile erection/Vaginal lubrication)	5 (3–5.5)	5 (4–6)	0.122 *
ASEX-4 (Ability to orgasm)	5 (4–5)	5 (2–6)	0.935 *
ASEX-5 (Satisfaction)	5 (3–5)	5 (3–5)	0.870 *
ESS Total Score	6 (3–11)	7 (4–12)	0.437 *
PSQI Total Score	6 (4–9)	7 (4–11)	0.305 *

*Mann–Whitney U test; **chi-square test. Categorical variables are presented as n (%), and continuous variables as median (interquartile range, IQR). PDQ-39: Parkinson's Disease Questionnaire; UPDRS: Unified Parkinson's Disease Rating Scale; H&Y: Hoehn and Yahr stage; ASEX: Arizona Sexual Experiences Scale; ESS: Epworth Sleepiness Scale; PSQI: Pittsburgh Sleep Quality Index.
